# Au nanorod assembly for sensitive SERS detection of airway inflammatory factors in sputum

**DOI:** 10.3389/fbioe.2023.1256340

**Published:** 2023-12-12

**Authors:** An-qi Yang, Wentong Zheng, Xiaoyang Chen, Jiayin Wang, Shuang Zhou, Hongzhi Gao

**Affiliations:** ^1^ Department of Basic Medicine, Quanzhou Medical College, Quanzhou, China; ^2^ Central Laboratory, Second Affiliated Hospital of Fujian Medical University, Quanzhou, China; ^3^ Clinic Laboratory, Dehua Hospital, Quanzhou, China; ^4^ Department of Pulmonary and Critical Intensive Medicine, Second Affiliated Hospital of Fujian Medical University, Quanzhou, China; ^5^ Department of Neurosurgery, Second Affiliated Hospital of Fujian Medical University, Quanzhou, China

**Keywords:** SERS (surface-enhanced Raman spectroscopy), biosensor, inflammatory factor, sputum, asthma

## Abstract

In this paper, we demonstrate a surface-enhanced Raman spectroscopy (SERS) biosensor based on the self-assembly of gold nanorods (AuNRs) for the specific detection of airway inflammatory factors in diluted sputum. The AuNR surface was modified with an antibody that was able to specifically recognize an airway inflammatory factor, interleukin-5 (IL-5), so that a end-to-end self-assembly system could be obtained, resulting in an order of magnitude amplification of the Raman signal and greatly improved sensitivity. Meanwhile, the outer layer of the biosensor was coated with silicon dioxide, which improved the stability of the system and facilitated its future applications. When the detected concentration was in the range of 0.1–50 pg/mL, the SERS signal generated by the sensor showed a good linear relationship with the IL-5 concentration. Moreover, it had satisfactory performance in diluted sputum and clinical subjects with asthma, which could achieve sensitive detection of the airway inflammatory factor IL-5. Overall, the developed biosensor based on the SERS effect exhibited the advantages of rapid and sensitive detecting performance, which is suitable for monitoring airway inflammatory factors in sputum.

## Graphical Abstract

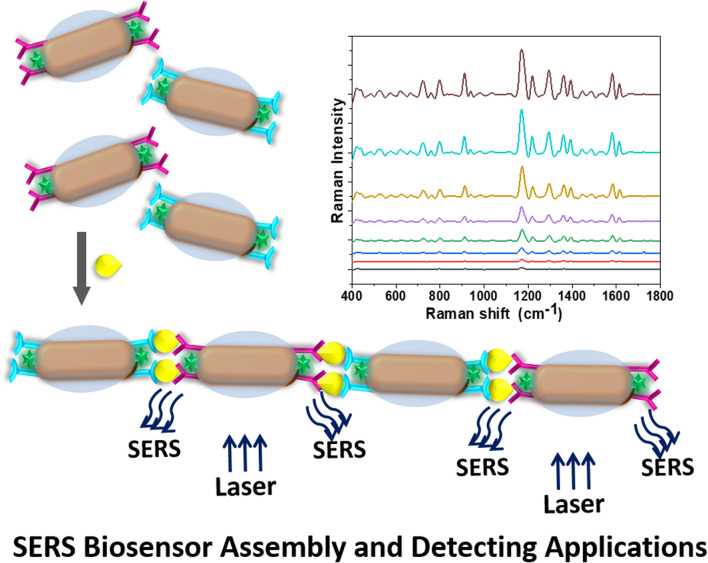

## 1 Introduction

Asthma, as one of the most common chronic respiratory diseases, is a long-term inflammatory disease involving the inflammatory cells, structural cells, and relevant cellular components of the airways (Dharmage et al., 2019). At present, both direct and indirect methods for monitoring airway inflammation in asthma have been applied to study asthma pathogenesis, disease assessment, and treatment response (Li, 2019; Hammad and Lambrecht, 2021). Direct methods, such as bronchoalveolar lavage fluid and bronchial mucosal biopsy, provide reliable results. However, these techniques bring certain risks and are limited by the progression of the disease. In addition, they are not suitable for short-term reuse, which limits their role as routine clinical detection methods (Gut et al., 2020; Lou et al., 2021). The relatively non-invasive sputum induction for cytological examination may lead to temporarily reduced lung function, and patients may experience symptoms such as chest tightness, cough, wheezing, or aggravated dyspnea. Also, it is time-consuming, expensive, and requires professional technicians to perform the procedure (Zong et al., 2022). Compared with induced sputum, sampling of natural sputum is non-invasive and easy to obtain. However, it has fewer inflammatory factors (biomarkers), which can lead to false negatives.

Currently, methods that are commonly used in laboratories mainly include radioimmunoassay (RIA), enzyme-linked immunosorbent assay (ELISA), chemiluminescence immunoassay (LICA), and electrochemiluminescence immunoassay (ECLIA) (Aitekenov et al., 2021; Momenbeitollahi et al., 2022). Despite their advantages, RIA is radioactive, which is harmful to human health; LICA requires high-purity reagents; and ECLIA requires complex operation and specialized equipment (Choi et al., 2013; Ma et al., 2020). ELISA, which is commonly used in clinical laboratory testing, also faces problems such as complicated and time-consuming operation, low sensitivity, and requiring certain locations and equipment (Zhu et al., 2012). Considering the challenge of inflammatory biomarker detection in sputum, it is necessary to develop a fast and simple biosensing system that is more sensitive and effective than traditional ELISA.

In recent years, nanoparticle assembly has been extensively studied due to its outstanding physical and chemical properties (Chen et al., 2020; Yin et al., 2022a). One of the important application directions is based on the “hot spots” generated after nanoparticle assembly to enhance the Raman signal by orders of magnitude (Yin et al., 2022b; Pillanagrovi and Dutta-Gupta, 2022). However, due to the instability of the assembly system, its potential for practical application is greatly limited (Zhong et al., 2011). In this work, we chose the key cytokine (interleukin-5, IL-5), which maintains the persistence of airway inflammation, as a detection biomarker (Walsh, 2020). By utilizing the anisotropy of gold nanorods (AuNRs), a sensitive nano-biosensor was prepared by modifying AuNRs with Raman-active molecules, silica, SH-PEG-COOH, and specific antibodies. Through the specific recognition between the airway inflammatory factor IL-5 and the antibody, gold nanorods were induced to form self-assemble end-to-end to generate hot spots. The Raman signal was thus amplified by an order of magnitude, maximizing the sensitivity of surface-enhanced Raman spectroscopy (SERS) and reducing the detection time. By coating the silica layer, the instability of the assembly system was greatly reduced, providing the potential for future applications to detect various types of biological samples. This study evaluates the stability, specificity, and recovery rate of the SERS self-assembly sensor in diluted sputum. Sputum IL-5 was measured by the ELISA kit and the SERS biosensor, respectively, in clinical subjects with asthma. The results demonstrate that the sensing system based on immune-induced self-assembly of AuNRs to enhance the SERS signal is an ideal approach for the detection of airway inflammatory factors.

## 2 Experimental section

### 2.1 Reagents and chemicals

Chloroauric acid (HAuCl_4_·3H_2_O, 99%), cetyltrimethylammonium bromide (CTAB, 99%), silver nitrate (AgNO_3_, >99%), sodium borohydride (NaBH_4_, 99%), L-ascorbic acid (AA, 99%), trisodium citrate (C_6_H_7_O_5_Na_3_), tetraethyl orthosilicate (TEOS), sodium hydroxide (NaOH), (3-dimethylaminopropyl) ethyl-carbodiimide monohydrochloride (EDC), malachite green isothiocyanate (MGITC), phosphate-buffered saline (PBS), N-hydroxysuccinimide (NHS), and 2-(N-morpholino) ethane sulfonic acid (MES) were purchased from Aladdin Chemical Reagent Co. Ltd., China. Thiolcarboxylic polyethylene glycol (HS-PEG-COOH, MW∼400), dithiothreitol (DTT), Interleukin 5 (IL-5), a monoclonal antibody to human IL-5 (detector antibody, dAb), a monoclonal antibody to human IL-5 (capture antibody, cAb), Interleukin 8 (IL-8), immunoglobulin E (IgE), and eosinophil cation protein (ECP) were purchased from Sangon Biotech Co. Ltd., China. Ultrapure water (18 MΩ cm^-1^) was used throughout the experiment.

### 2.2 Patients and sputum processing

Patients with asthma were recruited from the Second Affiliated Hospital of Fujian Medical University. Subjects with asthma (n = 10) had objective evidence of variable airflow obstruction as indicated by one or more of the following: 1) methacholine airway hyperresponsiveness (PC20 FEV1 < 8 mg/mL), 2) >15% improvement in FEV1 10 min after 200 mg inhaled salbutamol, or 3) peak expiratory flow (>20% maximum within-day amplitude from twice-daily peak expiratory flow measurements over a period of 14 days).

The protocol for sputum processing was modified from that recommended by Woolhouse et al. (2002) Sputum samples were treated with freshly prepared 0.1% DTT at a ratio of 4:1 (v/w). After treatment, each sample was rocked for 15 min. An additional 4 volumes of NaCl were added, which were then rocked for a further 5 min. The sputum was centrifuged at 1,200 rpm for 5 min and the supernatant was stored at −80°C until required.

### 2.3 Synthesis of AuNRs@SiO_2_

AuNRs were prepared by the seed-mediated growth process, according to the report by Sau and Murphy (2004). The Au seed solution was prepared by adding a freshly ice-cold NaBH_4_ solution (0.60 mL, 0.01 M) to a mixed aqueous solution composed of HAuCl_4_ (0.25 mL, 0.01 M) and CTAB (7.50 mL, 0.1 M). This seed solution was stirred rapidly for 2 min and then kept at room temperature (25°C) for 2 h. To prepare the AuNRs growth solution, HAuCl_4_ (4 mL, 0.01 M) and AgNO_3_ (0.75 mL, 10 mM) were mixed with CTAB (95 mL, 0.1 M), followed by the addition of a freshly prepared AA solution (0.64 mL, 0.1 M). In total, 0.4 mL of Au seed solution was added to the AuNRs growth solution with rapid stirring for 3 min, then kept at room temperature for 3 h. The resulting AuNRs were separated and purified by centrifugation at 10,000 rpm for 30 min.

AuNRs@SiO_2_ was prepared by the sol-gel method with a slight modification (Jiang et al., 2020). The prepared AuNRs solution was centrifuged at 7,500 rpm for 20 min and redispersed in ultrapure water. MGITC solution (100 μL, 10 μM) was added dropwise to mix well with AuNRs solution, centrifuged at 7,500 rpm for 15 min, and redispersed in 10 mL of ultrapure water. NaOH (0.1 mL, 0.1 M) was added to the mixture with stirring for 10 min 40 μL of TEOS/methanol (V/V: 1/4) solution was injected three times at 30-min intervals. The reaction was allowed to be gently stirred for 24 h, and the resulting colloidal solution was centrifuged twice at 4,500 rpm for 15 min.

### 2.4 Modification of the AuNRs@SiO_2_ biosensor

HS-PEG-COOH (20 μL, 20 μM) was added dropwise to mix well with the AuNRs@SiO_2_ solution, centrifuged at 4,500 rpm for 15 min, and redispersed in 1 mL of PBS. In order to activate the carboxyl groups, MES (50 μL, 0.25 M) and EDC (15 μL, 0.1 M) were added to the mixture and reacted for 20 min at room temperature with gentle inversion, followed by the addition of NHS (10 μL, 0.5 M). After mixing for 20 min, the mixture was purified by centrifugation at 4,500 rpm for 15 min and redispersed in 1 mL of PBS. Detection antibody (10 μL, 5 μg/mL) and capture antibody (10 μL, 5 μg/mL) were added to the mixture (500 μL). Both were stirred for 3 min at room temperature, followed by incubation at 37°C for 1 h. The two separate reaction parts were mixed together to obtain a biosensor solution for IL-5 detection.

### 2.5 SERS detection of IL-5

A total of 10 μL of IL-5 at different concentrations (0, 0.05, 0.1, 0.5, 1, 5, 10, and 50 pg/mL) were added to the biosensor solution and shaken at 37°C for 15 min. The mixtures were then tested by Raman spectroscopy. To examine the stability of the biosensor, IL-5 (10 μL, 10 μg/mL) was added to 1 mL of biosensor solution with 50% diluted serum and 50% diluted sputum, followed by incubation at 37°C for 15 min. The mixtures were then measured by Raman spectroscopy for 1 week. To examine the specificity of the biosensor, ECP, IgE, IL-8, and IL-5 (10 μL, 10 μg/mL) were added to 1 mL of biosensor solution with 50% diluted serum and 50% diluted sputum, followed by incubation at 37°C for 15 min. The mixtures were then measured by Raman spectroscopy. To evaluate the response of the biosensor, 10 μL of IL-5 with different concentrations (0.5, 1, 5, and 10 pg/mL) were added to 1 mL of biosensor solution with 50% diluted sputum, followed by incubation at 37°C for 15 min. The samples were then analyzed by Raman spectroscopy.

### 2.6 Measurement of IL-5 in sputum samples

Sputum IL-5 was measured by ELISA (Shanghai Jianglai Industrial Limited by Share Ltd., Shanghai, China). The concentration detection range was 1.56–100 pg/mL. The detection limit was 0.38 pg/mL. Sputum IL-5 was measured by SERS. In total, 1 mL of sputum supernatant was added to 1 mL of biosensor solution, followed by incubation at 37°C for 15 min. The samples were then analyzed by Raman spectroscopy.

### 2.7 Characterization

Transmission electron microscopy (TEM) images were obtained using a JEM-1400 (100 kV) microscope. The UV-vis absorption spectra were obtained using a TU-1810 UV-vis spectrophotometer. The zeta potential was measured with a Malvern Zetasizer ZEN3600 instrument. Energy-dispersive X-ray spectroscopy (EDS, Thermo Scientific Talos F200X) was used to analyze the composition. Raman spectra were obtained using a compact Raman system with 785 nm excitation and a 1 s exposure time (40 mW, Advantage Raman Series, DeltaNu).

### 2.8 Calculation of the SERS enhancement factor

The surface enhancement factor (*EF*) was calculated using Eq. 1 (Dong et al., 2021).

$$EF=\frac{I_{SERS}/N_{SERS}}{I_{NRS}/N_{NRS}}=\frac{I_{SERS}/\left(C_{SERS}\times V_{SERS}\right)}{I_{NRS}/\left(C_{NRS}\times V_{NRS}\right)}=\frac{I_{SERS}/C_{SERS}}{I_{NRS}/C_{NRS}}$$ (1)

where *I*_*SERS*_ is the intensity of the SERS spectrum of the MGITC-labeled biosensor assembly at 1,172 cm^−1^, and *I*_*NRS*_ is the intensity of the same peak of the normal Raman spectrum of the MGITC aqueous solution. *N*_*NRS*_ and *N*_*SERS*_ represent the number of MGITCs irradiated by a laser beam in normal Raman scattering (NRS) and SERS, respectively. *V*_*NRS*_ and *V*_*SERS*_ are the volumes of solution excited by a laser beam in NRS and SERS, respectively. *C*_*NRS*_ is the concentration of MGITC in the water solution. *C*_*SERS*_ denotes the concentration of MGITC in the SERS biosensor solution. The strongest Raman intensity at 1,172 cm^−1^ peak was chosen for EF calculation.

The rate of the surface enhancement factor (REF) was calculated based on Eq. 2: (Lee et al., 2021):

$$R_{\text{EF}}=\frac{{\text{EF}}_{\text{Assembly}}}{{\text{EF}}_{Single}}=\frac{\frac{I_{SERS^{Assembly}}/N_{SERS^{Assembly}}}{I_{NRS}/N_{NRS}}}{\frac{I_{SERS^{single}}/N_{SERS^{single}}}{I_{NRS}/N_{NRS}}}=\frac{I_{SERS^{Assembly}}}{I_{SERS^{single}}}$$ (2)

where *EF*_*Assembly*_ is the EF of the SERS spectrum of the MGITC-labeled biosensor assembly, and EF_Single_ is the EF of the same peak of the normal Raman spectrum of the biosensor single solution.

## 3 Results

### 3.1 Characterization of AuNRs and AuNRs@SiO_2_

**FIGURE 1:**
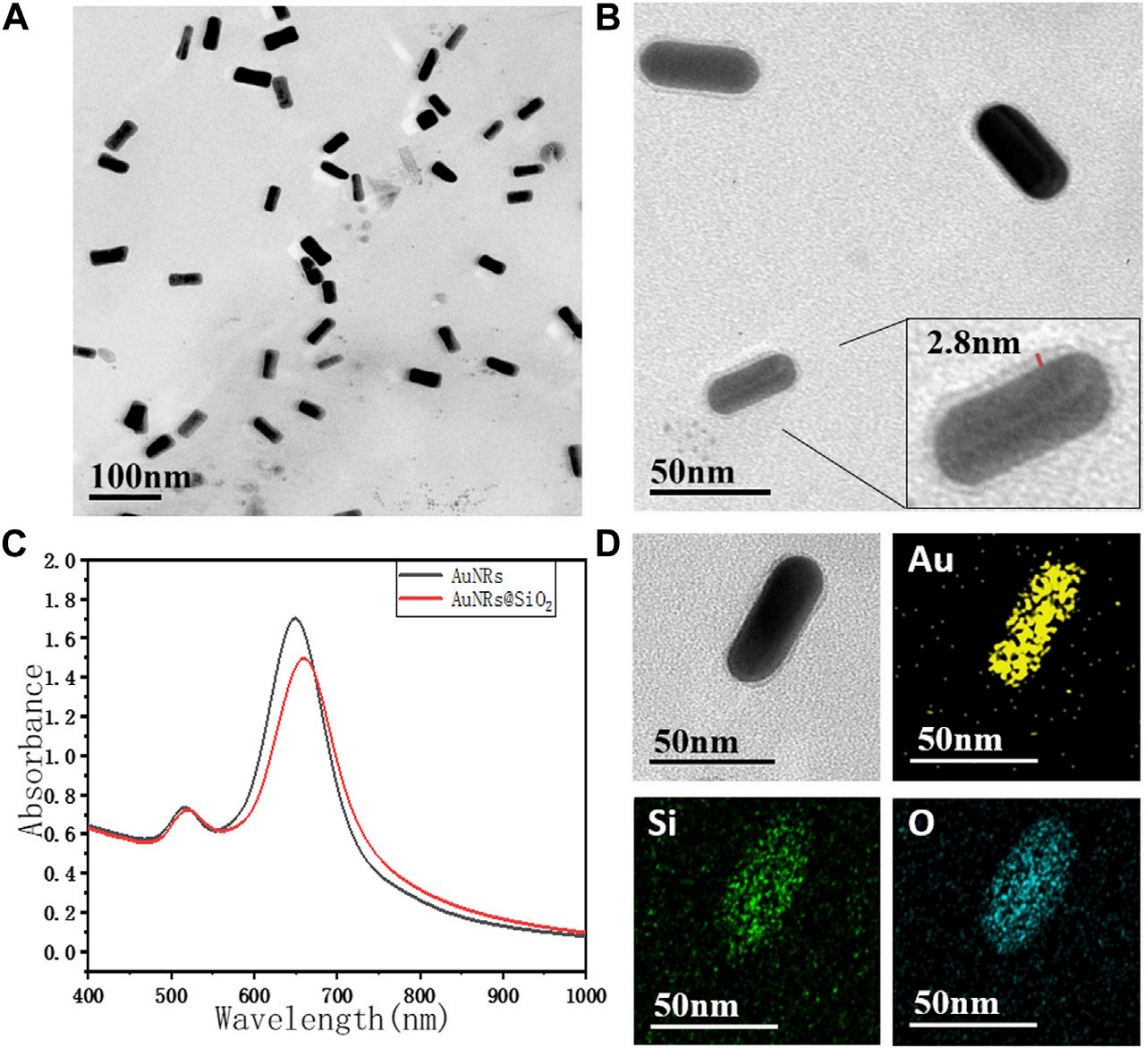
The TEM image of **(A)** AuNRs, **(B)** AuNRs@SiO_2,_ and **(C)** the UV-vis spectra. TEM-EDS images **(D)** of AuNR@SiO_2_, and the chemical mapping of Au, Si, and O are shown.

As shown in Figure 1A, the AuNRs prepared by the seeded growth method exhibited uniform size and good monodispersity. The length of the long axis was 55 ± 5 nm, and the short axis length was 20 ± 2 nm, with an aspect ratio of 2.75 ± 0.5. As shown in Figure 1B, the silicon dioxide layer of AuNRs@SiO_2_ was extremely thin (2∼3 nm). However, it did not completely cover the surface of the nanorods: the silicon dioxide layer on the side of the gold nanorods was more obvious, while almost no coating was observed on the head. The UV-Vis absorption spectrum of AuNRs@SiO_2_ is shown in Figure 1C. It can be seen from the figure that the AuNRs had two obvious peaks of surface plasmon resonance (SPR): 516 nm (SPR on the horizontal axis) and 650 nm (SPR on the vertical axis). After the AuNRs were coated with silica, the SPR in the horizontal axis direction was maintained, while the SPR peak in the vertical axis direction showed a red shift to 660 nm.

Since the preparation of AuNRs requires the use of a high concentration of CTAB as a surfactant to maintain the stability of the nanorods, these AuNRs tend to aggregate with the reduction of CTAB during the subsequent modification process. Moreover, CTAB has strong biological toxicity, which greatly limits the application potential of AuNRs in the fields of biological research, medical imaging, and treatment. Therefore, in this paper, SiO_2,_ which has better biocompatibility, was used for surface modification. AuNRs@SiO_2_ could not only improve the stability and dispersibility of the nanoparticles but also have excellent light transmittance. The coated AuNRs would have less impact on the light-absorbing properties and could also provide a large number of connection sites, making the subsequent modification and biomolecule coupling much easier.

It is worth noting that the thickness of the silica layer was found to be highly related to the thickness of the CTAB layer on AuNRs. TEOS can be easily attracted by positively charged CTAB during the hydrolysis process, so that the TEOS is hydrolyzed and coated on the surface of AuNRs (Pérez-Juste et al., 2005). Through the seed-mediated growth method, the {100} planes on the side of AuNRs were generally wrapped by a thick layer of CTAB, while the {110} planes on the head were relatively less (Hill et al., 2009). Thus, when a small amount of TEOS was added, the TEOS was preferentially attracted to the side of AuNRs for hydrolytic coating. In contrast, the head of AuNRs had less CTAB, so it could not attract enough TEOS, which made it difficult to form a silicon dioxide layer.

This work then suggests the use of EDS spectroscopy to explain how SiO_2_ is applied to AuNRs (Figure 1D). The results displayed in the figure, which shows the distribution of Au, Si, and O elements, demonstrate that the distribution ranges of these elements remain mostly unchanged along the length of the AuNRs. However, across the width of the AuNRs, the distribution ranges of Si and O elements are wider than those of Au. This suggests that SiO_2_ primarily coats the sides of the AuNRs, while there is less or no coating at their ends. As a result, the heads of the AuNRs are comparatively bare for the upcoming synthetic modification. This suggests that there is more SiO_2_ encapsulation on the sides of the AuNRs and less or no encapsulation on the end of the AuNRs.

### 3.2 Immune-induced self-assembly of AuNRs

As mentioned above, during the preparation of AuNRs@SiO_2_, a small amount of SiO_2_ was preferentially coated on the sides of the AuNRs, while the heads were relatively bare. Therefore, when the bifunctional molecule SH-PEG-COOH was added, it tended to couple to the heads of the AuNRs through the strong Au-S chemical bonds. Furthermore, to modify the IL-5 antibody on the AuNRs, the carboxyl groups in SH-PEG-COOH needed to be activated. With the activation by EDC-NHS, the carboxyl group of SH-PEG-COOH was chemically bonded with the amino group on the surface of the antibody, forming a stable amide bond (Yuan et al., 2021). Thus, the IL-5 antibody was attached to the heads of the AuNRs. In the construction of an anti-IL-5-coated AuNR biosensor, when IL-5 antigen was added, the specific recognition could trigger the end-to-end self-assembly of the AuNRs.

**FIGURE 2:**
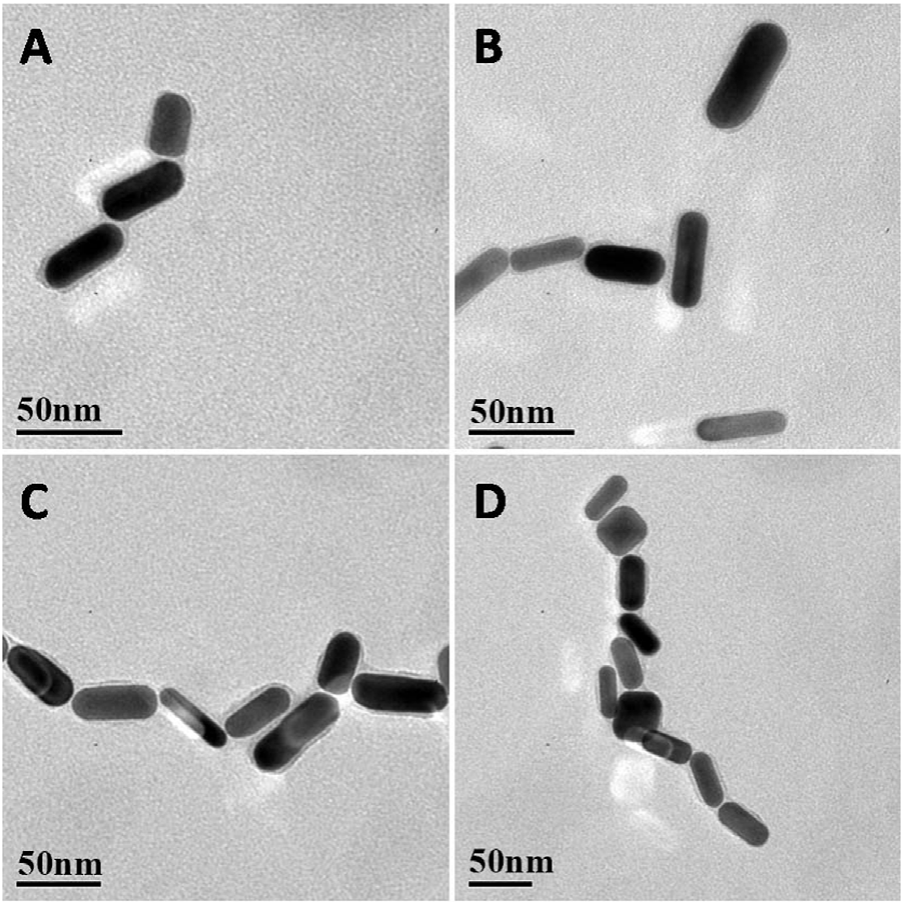
**(A–D)** Representative TEM images of AuNRs biosensor EE assembly formed with the addition of different concentrations of IL‐5 0.1, 1, 10, 50 pg/mL.

As shown in Figure 2, when IL-5 solution was added to the AuNR sensor, the AuNRs formed an ordered structure by end-to-end self-assembly. At a low concentration of IL-5 (e.g., 0.1 pg/mL), the IL-5 antigen was not able to bind all the AuNRs (Figure 2A), so the self-assembled structure was shorter. With the increase in IL-5 concentration, the end-to-end self-assembled structure gradually elongated. When the other conditions remained unchanged, the number of end-to-end structures was directly related to the concentration of IL-5 added, indicating that the formation of the self-assembled AuNR structure was caused by the specific binding between IL-5 and antibody.

### 3.3 Study on the SERS activity of the prepared AuNRs

**FIGURE 3:**
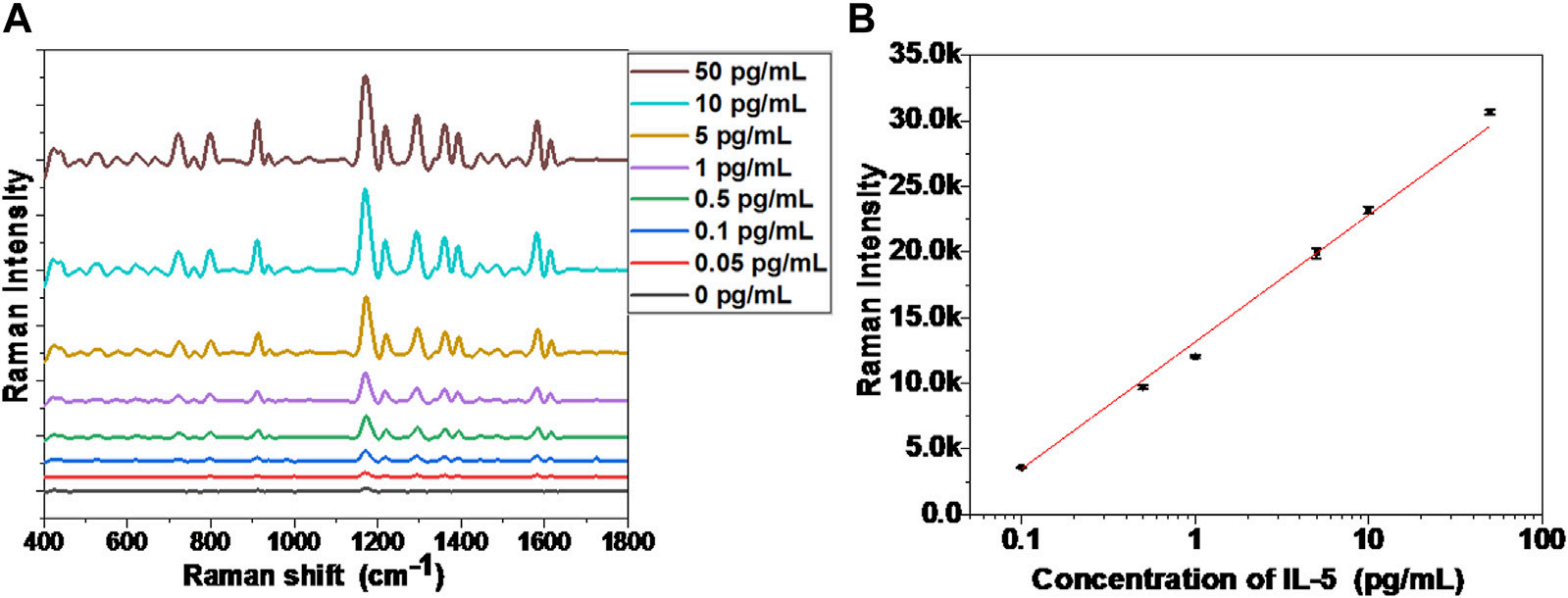
**(A)** SERS spectra of the biosensor incubated with different concentrations of IL-5, **(B)** The calibration curve between Raman intensity (1,172 cm^−1^ peak) and IL-5 concentration.

Under an appropriate light wavelength, AuNRs underwent a plasmon coupling effect to enhance the surrounding electromagnetic field, which exhibited the SERS effect (Dey et al., 2020). When the AuNRs formed a self-assembled structure, the electromagnetic field near the gap of the AuNRs was further enhanced, so that the Raman signal was amplified by an order of magnitude (Xie et al., 2019). The Raman-active molecule selected in this study was isothiocyanate malachite green (MGITC), which contains several characteristic fingerprint peaks in the Raman spectrum: 798, 912, 1,172, 1,295, 1,362, and 1,583 cm^−1^ (Kang et al., 2020). In order to illustrate the sensitivity of the MGITC-labeled AuNR biosensor, different concentrations of IL-5 were added for testing, and the SERS spectrum was collected as shown in Figure 3A. When the concentration of IL-5 was 0 pg/mL, the sensor was not yet assembled, and the corresponding SERS signal in Figure 3A was very weak, showing only the SERS signal of MGITC in monodispersed AuNRs. With the increase of IL-5 concentration, the end-to-end self-assembly of AuNRs was triggered. The Raman signal of MGITC was gradually enhanced and amplified by orders of magnitude due to the SPR effect between adjacent AuNRs. It is worth noting that the number of assemblies increased with the increase in IL-5 concentration, which was consistent with the results in Figure 2. When the concentration of IL-5 was 50 pg/mL, the SERS signal was the strongest. The calculation formulas for EF and R_EF_ were then obtained. As a result, the EF, in this case, was 1.39 × 10^5^, and the R_EF_ was 33.1. When the IL-5 concentration was 0.05 pg/mL, the corresponding SERS signal was only slightly higher than that of the monodisperse AuNRs, and the EF and R_EF_ values were 6.21 × 10^3^ and 1.48, respectively. Therefore, the AuNR biosensor was able to effectively enhance the Raman signal of MGITC by inducing the formation of end-to-end self-assembly through biorecognition.

As shown in Figure 3B, the relationship curve between the SERS signal and IL-5 concentration was presented, where the SERS signal was obtained by taking the strongest and characteristic Raman peak of MGITC at 1,172 cm^−1^. By linear fitting, the SERS signal showed a good linear relationship with the detected IL-5 concentration in the range of 0.1–50 pg/mL, after end-to-end self-assembly of AuNRs. The equation for the calibration curve can be described as follows:

Y=9665.57 X+13164.96 (3)

where Y is the SERS signal intensity of 1,172 cm^−1^, and X represents the logarithmic value of IL-5 concentration. The correlation coefficient (*R* ^2^) of the equation is 0.994. In this paper, the linear range of the AuNR sensor for IL-5 concentration detection was 0.1–50 pg/mL, far lower than the detection range of ELISA detection kits commonly used for IL-5 detection (Tanaka et al., 2020; Yu and Li, 2022). Thus, the self-assembly of AuNRs with the enhanced SERS signal could be used to detect airway inflammatory factors, and its detection range could fully meet the requirements of clinical applications.

### 3.4 Performance Evaluation of the Biosensor

**FIGURE 4:**
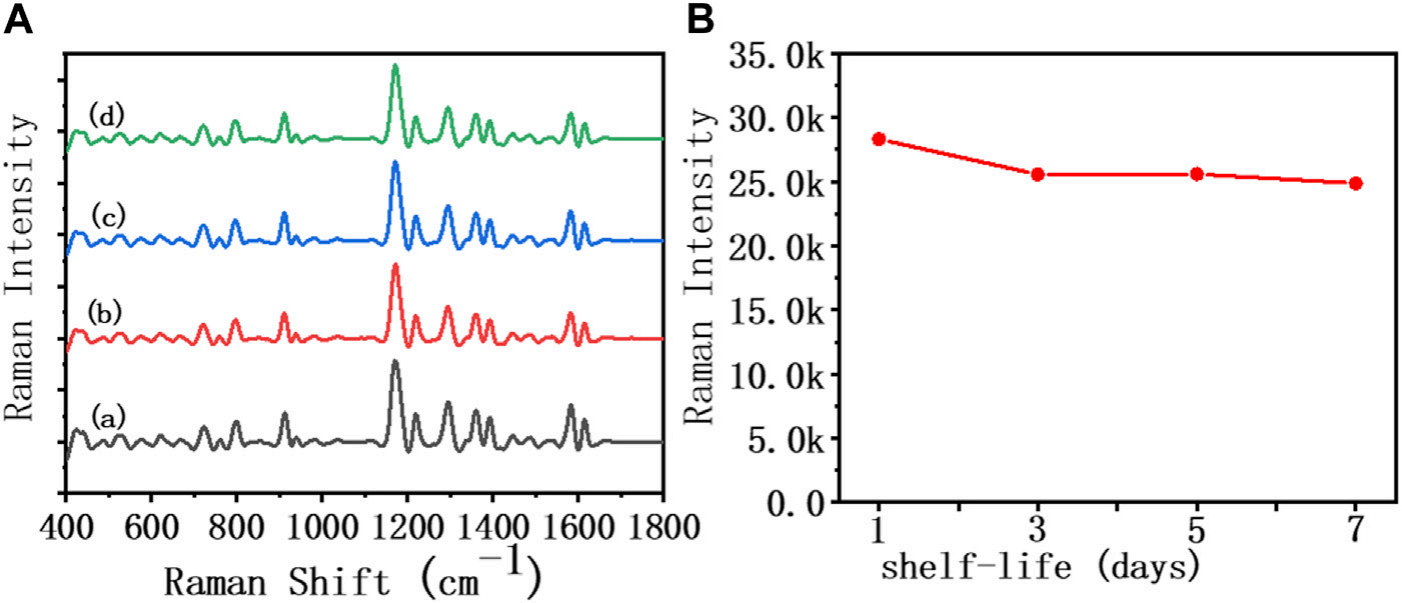
SERS spectra and biosensor incubated in an IL-5 (50 pg/mL) solution for 1 week in **(A)** 50% diluted sputum sample, **(B)** the Raman intensity at 1,172 cm^−1^ for 1 week.

In order to further explore the feasibility of the developed AuNR biosensor in clinical applications, we investigated the stability, specificity, and recovery rate of this method in diluted sputum, respectively. As shown in Figure 4, the SERS spectrum was recorded, reflecting the changes in the characteristic peaks of MGITC within 1 week of the assembly system formed by IL-5-induced specific recognition. Within the first 3 days, the intensity of the characteristic peak at 1,172 cm^−1^ in 50% diluted sputum decreased by 9.7% from 28,317 to 25,559. In the following days, the MGITC characteristic peaks showed little change, and the assembly system remained unchanged. The results indicated that the sensor prepared in this paper had good stability in diluted sputum.

**FIGURE 5:**
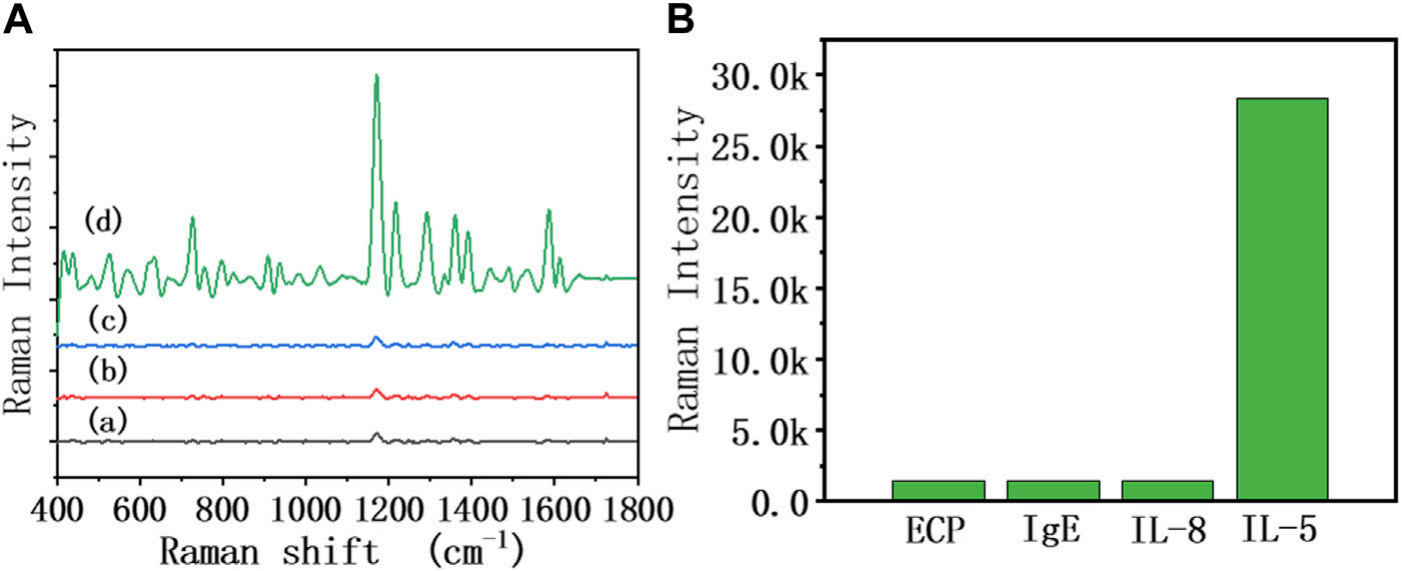
**(A)** SERS spectra of the 50% diluted sputum group of the biosensor in the presence of different interference reagents: (a) ECP, (b) IgE, (c) IL-8, and (d) IL-5 at the same concentration (50 pg/mL), **(B)** The relationship between Raman intensity (1,172 cm^−1^ peak) and different interference reagents.

As shown in Figure 5, different interference reagents and IL-5 were spiked into the sensor at 50 pg/mL, and the SERS spectra were collected. It can be seen that the SERS signal of added IL-5 was significantly enhanced, while the rest of the interference reagents showed no obvious change in the SERS signal. This result showed that the biosensor had good specificity. The antibody modified on the heads of the AuNRs showed specific biorecognition of IL-5 and did not bind with the interference reagent.

As shown in Table 1, a recovery rate experiment with the biosensor was also carried out. The intensity of the highest characteristic peak at 1,172 cm^−1^ in the SERS spectrum was collected. The concentration of IL-5 could be obtained using Eq. 3, and the recovery rate of the detection method was calculated. In 50% diluted sputum, the method yielded IL-5 recoveries in the range of 94.3%–109.4%. These results showed that the AuNR biosensor still maintained good sensitivity in complex biological environments such as sputum, which would fulfill the sensitive detection of trace airway inflammatory factors.

**TABLE 1:** Recoveries of the known spiked amounts of IL-5 in a 50% diluted sputum sample (pg/mL).

*Sample No.*	*1*	*2*	*3*	*4*	*5*
IL-5 Addition	0.5	1	5	10	25
IL-5 Detection	0.547	0.961	5.24	9.43	24.61
Recovery (%)	109	96.1	104.8	94.3	98.4

### 3.5 Measurement of IL-5 in sputum samples

In this study, sputum IL-5 was measured by an ELISA kit and SERS biosensor in 10 subjects with asthma. As shown in Table 2, there are three cases that tested negative for the ELISA, and the agreement between the two methods was good for the other cases. The sputum IL-5 of samples 1, 4, and 5 measured by the SERS biosensor were 0.91, 0.68, and 1.32 pg/mL, respectively, which were not in the detection range of the ELISA kit (1.56–100 pg/mL). Compared to the ELISA method, the SERS biosensor provided greater accuracy and higher detection sensitivity.

**TABLE 2:** Results of IL-5 detection using two methods (pg/mL).

Detection methods	Sputum samples									
	1	2	3	4	5	6	7	8	9	10
SERS biosensor	0.91	5.66	10.45	0.68	1.32	11.48	8.75	-	14.92	8.47
ELISA Kit	-	4.97	11.12	-	-	11.63	9.22	-	15.84	7.69

## 4 Conclusion

In this paper, the immune-induced self-assembly of AuNRs was developed to enhance the SERS signal, and the established system was applied to realize the sensitive detection of trace airway inflammatory factors in diluted sputum. Utilizing the characteristics of AuNRs, the Raman-active molecule MGITC, silicon dioxide, and specific antibodies were sequentially modified on the AuNR surface to form sensitive and stable nano-biosensors. Through the specific recognition between IL-5 antigen and antibody, the end-to-end self-assembly of AuNRs was induced, resulting in the enhancement of the SERS signal. By coating the SiO_2_ layer, the stability of the assembly system was greatly improved, which would be beneficial for future applications. When the detected concentration was in the range of 0.1–50 pg/mL, the SERS signal showed a good linear relationship with the detected IL-5 concentration. In addition, the biosensor maintained good stability, specificity, and sensitivity in diluted sputum. Compared with the traditional ELISA method, the developed method showed the advantages of higher sensitivity, simple operation, and short detection time in clinical subjects with asthma. Thus, it is an ideal approach to detecting airway inflammatory factors and has great value in airway inflammation monitoring and clinical diagnosis.

## Data Availability

The original contributions presented in the study are included in the article/Supplementary Material, further inquiries can be directed to the corresponding author.
